# Socioeconomic Inequity in the Screening and Treatment of Hypertension in Kenya: Evidence From a National Survey

**DOI:** 10.3389/frhs.2022.786098

**Published:** 2022-04-05

**Authors:** Robinson Oyando, Edwine Barasa, John E. Ataguba

**Affiliations:** ^1^Health Economics Research Unit, KEMRI-Wellcome Trust Research Programme, Nairobi, Kenya; ^2^Health Economics Unit, Faculty of Health Sciences, School of Public Health and Family Medicine, University of Cape Town, Cape Town, South Africa; ^3^Center for Tropical Medicine and Global Health, Nuffield Department of Medicine, University of Oxford, Oxford, United Kingdom; ^4^Department of Community Health Sciences, Rady Faculty of Health Sciences, Max Rady College of Medicine, University of Manitoba, Winnipeg, MB, Canada

**Keywords:** socioeconomic inequality, horizontal inequity, decomposition analysis, hypertension, Kenya

## Abstract

**Background:**

Non-communicable diseases (NCDs) account for 50% of hospitalisations and 55% of inpatient deaths in Kenya. Hypertension is one of the major NCDs in Kenya. Equitable access and utilisation of screening and treatment interventions are critical for reducing the burden of hypertension. This study assessed horizontal equity (equal treatment for equal need) in the screening and treatment for hypertension. It also decomposed socioeconomic inequalities in care use in Kenya.

**Methods:**

Cross-sectional data from the 2015 NCDs risk factors STEPwise survey, covering 4,500 adults aged 18–69 years were analysed. Socioeconomic inequality was assessed using concentration curves and concentration indices (CI), and inequity by the horizontal inequity (HI) index. A positive (negative) CI or HI value suggests a pro-rich (pro-poor) inequality or inequity. Socioeconomic inequality in screening and treatment for hypertension was decomposed into contributions of need [age, sex, and body mass index (BMI)] and non-need (wealth status, education, exposure to media, employment, and area of residence) factors using a standard decomposition method.

**Results:**

The need for hypertension screening was higher among poorer than wealthier socioeconomic groups (*CI* = −0.077; *p* < 0.05). However, wealthier groups needed hypertension treatment more than poorer groups (*CI* = 0.293; *p* <0.001). Inequity in the use of hypertension screening (*HI* = 0.185; *p* < 0.001) and treatment (*HI* = 0.095; *p* < 0.001) were significantly pro-rich. Need factors such as sex and BMI were the largest contributors to inequalities in the use of screening services. By contrast, non-need factors like the area of residence, wealth, and employment status mainly contributed to inequalities in the utilisation of treatment services.

**Conclusion:**

Among other things, the use of hypertension screening and treatment services in Kenya should be according to need to realise the Sustainable Development Goals for NCDs. Specifically, efforts to attain equity in healthcare use for hypertension services should be multi-sectoral and focused on crucial inequity drivers such as regional disparities in care use, poverty and educational attainment. Also, concerted awareness campaigns are needed to increase the uptake of screening services for hypertension.

## Introduction

Non-communicable disease-related morbidity and mortality pose an increasing challenge globally, especially in low-and middle-income countries (LMICs), where most of the world's population live (1). In 2016, for instance, ~40 million deaths globally were due to non-communicable diseases (NCDs) with LMICs accounting for 80% of the deaths (2). LMICs also continue to struggle in containing the relatively high disease burden from maternal and child ill-health and infectious diseases such as HIV/AIDS, tuberculosis, leading to a “double burden” of communicable and NCDs (3, 4). This not only poses further resource constraints to the already overstretched healthcare resources in LMICs but is also a threat to the attainment of equity in health and healthcare between and within countries (5–7).

The major NCDs—cardiovascular diseases (CVDs), cancers, chronic respiratory diseases and diabetes—present a unique challenge to the global health agenda of attaining universal health coverage (UHC)^1^ by 2030 (1). Furthermore, the detrimental health, economic, and developmental consequences of NCDs have seen their inclusion in the 2030 Sustainable Development Goals (SDGs) (8). SDG 3.4 explicitly aims to reduce by one-third premature mortality due to NCDs through prevention and treatment. Prioritising the reduction of shared NCDs risk factors such as physical inactivity, unhealthy diets, use of tobacco, and harmful use of alcohol is imperative to achieve the SDGs (9). Similarly, for hypertension which is a major risk factor for CVDs (1), evidence shows that increasing access to preventive interventions such as timely screening among those at risk and providing treatment to those diagnosed are cost-effective measures of attaining the NCD pre-mature mortality target (10–12).

A well-functioning health system should ensure equity in the utilisation of health services, that is, based on need and not the ability to pay (13, 14). Yet, there is convincing evidence that the poor (who bear the greatest NCDs burden and are most in need of screening and treatment) relative to the rich, utilise NCDs healthcare services the least (15–19). This phenomenon is termed *the inverse care law* (20). Demand and supply-side factors such as high levels of poverty, the substantial economic burden associated with the long-term care of NCDs, and insufficient health system capacity to handle NCDs (chiefly at the primary care level) are some of the reasons that contribute to socioeconomic inequalities in NCDs (1, 3, 15, 17, 21–23).

Empirical evidence from previous studies that assessed inequity and socioeconomic inequality in hypertension converge to the same conclusion: that the poor, relative to the wealthy, utilise fewer hypertension services (15, 17, 18, 24, 25). Elwell-Sutton et al. (15), for instance, showed marked pro-rich inequality in the utilisation of treatment services for hypertension and dyslipidaemia in China. In addition, pro-rich horizontal inequity in the utilisation of hypertension, hyperglycaemia and dyslipidaemia treatment were reported in the same study (15). Of interest, income and other non-need factors (i.e., health insurance, education and longest-held occupation) mainly explained the observed inequality in NCDs treatment. These findings compare well with studies from other LMICs, which generally show that income, area of residence, level of education, occupational class, increasing age and lifestyle risk factors are significant contributors to the socioeconomic inequality in the prevalence or utilisation of NCD services (18, 25, 26).

In Kenya, NCDs account for 50% of hospitalisations and 55% of inpatient deaths, with estimates indicating that mortality due to NCDs is likely to increase by over 50% in the next decade (27). Besides, there are stark disparities in screening and treatment services utilisation for hypertension, mainly to the disadvantage of poorer socioeconomic groups. For example, 73% of the poorest quintile population have never been screened for hypertension compared to 38% in the richest quintile (27). Furthermore, a study in Kenya that estimated socioeconomic inequalities in hypertension prevalence found that the poor bore the highest burden, with body mass index (BMI), wealth status and education level mainly explaining the observed inequalities (24).

Although there is evidence suggesting inequalities in NCDs in Kenya, there is still a gap in knowledge, especially in assessing horizontal equity (i.e., equal treatment for equal need) in utilising screening and treatment for NCDs based on need. Also, to our knowledge, no study has assessed the factors contributing to socioeconomic inequality in using both interventions for hypertension in the Kenyan context. Therefore, using a nationally representative NCDs risk factors survey data set, this study aims to assess horizontal inequity in the screening and treatment of hypertension and decompose socioeconomic inequalities in the screening and treatment of hypertension in Kenya.

## Methods

### Data

This paper used the most recent and nationally representative cross-sectional STEPwise survey (STEPs) conducted by the Kenya National Bureau of Statistics (KNBS) between April and June 2015 in the country's 47 counties (27). The survey used the fifth National Sample Survey and Evaluation Programme (NASSEP V) master sample frame developed by the KNBS. The sample frame was developed using the Enumeration Areas (EAs) generated from the 2009 Kenya Population and Housing Census to form 5,360 clusters split into four equal sub-samples. A three-stage cluster sample design was used to collect the STEPs data. In the first stage, a total of 200 clusters (100 rural and 100 urban) were selected systematically from the NASSEP V sampling frame using the equal probability selection method to ensure the resulting sample retained the properties of probability proportional to size as was used in the creation of the frame. The second sampling stage involved a uniform selection of 30 households from the listed households in each cluster. An eligible participant was randomly selected from listed household members in the third sampling stage (27).

A total of 6,000 households were identified, but 4,754 consented (i.e. 79.2% response rate) and participated in the study. A total of 4,500 households were retained after data cleaning. A more detailed description of the STEPs data collection methodology is contained elsewhere (27). Sample weights were included in the statistical analyses to ensure that estimates were nationally representative. De-identified data set from the STEPs survey (which is available upon request from KNBS) was used in this study. Additionally, ethics clearance was obtained from the Human Research Ethics Committee of the University of Cape Town (Ref: 186/2020).

### Measuring Socioeconomic Status

Socioeconomic status (SES) can be measured using several approaches classified as “direct measures,” that is, expenditure, income, consumption; and “proxy measures,” including education, occupation or social class, but mainly asset indices (28). It is important to note that there are debates on the right choice of SES measure regarding health inequality assessment. Some argue that the choice of welfare measure may not overly affect inequality findings (29, 30) while others maintain that the computed health inequality results could be sensitive, in some contexts, to the choice of welfare measure (28, 31). Following similar studies (17, 32) and based on data availability, principal component analysis (PCA) (33) was used in this paper to generate an index of SES.

Briefly, the multivariable statistical approach (PCA) reduces the number of variables in a data set into smaller dimensions (34). Put another way, beginning with an initial set of correlated variables, PCA generates uncorrelated components, in which case each component or index is a linear weighted combination of the original variables (33). The first principal component provides what is needed to construct a household welfare index–if it explains a substantial proportion of the variance, with larger weights assigned to assets that vary most across households (29, 34). Data on 15 selected variables (e.g. source of drinking water, type of sanitary facility, roof, floor and wall material, source of energy for cooking and lighting, and ownership of TV, radio, refrigerator, washing machine, bicycle, motorcycle, landline, and cell phone) were used to generate standardised weighted scores. These variables were used to create a dummy of each variable, signifying the presence of each item given that categorical variables are converted into a meaningless scale in PCA (35). The composite weighted index was used to rank the sample into five wealth quintiles (1—poorest, 5—richest).

### Defining Hypertension

Having hypertension was defined in this paper based on any or all the three criteria: (1) previous hypertension diagnosis by a health worker, (2) use of prescribed anti-hypertensive medication or (3) having a systolic and/or diastolic blood pressure of ≥ 140/≥ 90 mmHg (36).

### Measuring Need and Use of Hypertension Screening and Treatment

The need for hypertension *screening* was defined as individuals who smoke, are obese (≥ 30 kg/m^2^) and are 30 years and above (for both men and women), as stipulated in Kenya's cardiovascular treatment guidelines (36). The need for hypertension *treatment* was defined as those diagnosed with hypertension in the survey (i.e. a third systolic and/or diastolic blood pressure of ≥ 140/≥ 90 mmHg, respectively) (36).

The utilisation of screening services was assessed as having ever received a screening service for hypertension from a formal health provider (i.e. doctor or other health workers) before the survey. Similarly, utilisation of treatment was assessed as taking prescribed hypertension treatment two weeks before the survey. For a granular presentation of inequality findings, the share of need and use of screening and treatment interventions were compared across the SES groups and regional divides in Kenya. Table 1 further summarises the definitions of variables used in the analysis.

**Table 1:** Definitions of variables used in the analysis.

**Intervention**	**NCD**	**Need**	**Use**
Screening	Hypertension	Respondents who are obese (≥30 kg/m^2^), smoke and are aged at least 30 years and have not been screened in the past	Respondents reporting ever screened by a health worker
Treatment	Hypertension	Respondents diagnosed with hypertension in the survey (i.e. systolic and/or diastolic blood pressure reading ≥ 140 mmHg or ≥ 90 mmHg)	Respondents reporting the use of prescribed anti-hypertensive treatment at least two weeks before the survey

### Analytical Approaches

#### Measuring Inequality in Care Utilisation

Inequality in screening and treatment can be assessed using various methodological approaches, as discussed by Wagstaff et al. (37). This paper used the concentration curves and concentration indices to assess inequality in the screening and treatment of hypertension. The rationale for using these measures is their consistency in ranking individuals according to their SES; sensitivity to changes in population distribution across SES, and ability to assess relative vs. absolute inequality (37–39). The concentration curve (CC) plots the cumulative share of the use of screening or treatment services (y-axis) against the cumulative share of households, ranked from poorest to richest (x-axis). So, if everyone uses screening or treatment services irrespective of their SES rank, the CC will consistently lie on the equality (45-degree) line. If, by contrast, there is a pro-poor (pro-rich) distribution in the use of screening or treatment services, the CC will lie above (below) the line of equality, with the gap between the CC and equality line depicting the extent of inequality (40).

The concentration index (CI_H_) was computed as twice the covariance between screening or treatment for hypertension and an individual's socioeconomic rank divided by the mean of the health variable. Theoretically, the CI_H_ lies between −1 (i.e. when the use of screening/treatment is concentrated on the poorest individual) and +1 (i.e. when the use of screening/treatment is concentrated on the richest individual). Overall, a positive (negative) CI_H_ corresponds to a pro-rich (pro-poor) distribution. For a binary variable, the concentration index does not lie within the usual bounds but rather between (μ_H_ - 1) and (1- μ_H_) and thus requires normalisation (41). Although there is a debate between Wagstaff (41–43) and Erreygers (44, 45) regarding the appropriate normalisation approach, this paper used the Wagstaff's (41) normalisation primarily because the health variable of interest was binary (i.e. 1 = use of screening/treatment; 0 = otherwise).

### Decomposing the Concentration Index of Screening and Treatment

While the CC and the CI_H_ are relevant in examining the existence of socioeconomic inequalities in screening/treatment; they do not explain the factors contributing to observed inequality. Consequently, to understand the factors contributing to relative inequality, the CI_H_ was further decomposed following the methodology suggested by Wagstaff et al. (46). Identifying these factors is critical for policy decisions around addressing the “underlying causes of inequality.” Thus, CI_H_ can be decomposed as:


(1) $$CI_{H}=\underset{Deterministic}{\underset{︸}{\sum_{j=1}^{J}C_{j}\left(\beta_{j}{\bar{Z}}_{j}/\mu_{H}\right)\;}}\;+\;\underset{Unexplained}{\underset{︸}{\left(GC\varepsilon /\mu_{H}\right)}}$$

where *Cj* (${\bar{Z}}_{j}$) is the concentration index (mean) of the jth contributing factor, *GCε* is the generalised concentration index of the error term (ε) and β_*j*_ is obtained from the linearly additive equation related to the contributing factors (z) to the screening or treatment variable (h) shown in Equation 2.


(2) $$\begin{array}{r} h_{i}=\alpha +\underset{j}{\sum }\beta_{j}Z_{ij}+\;\varepsilon_{i} \end{array}$$

where α and β_*j*_ are the coefficients to be estimated and ε_*i*_ is the error term. The deterministic portion of the concentration index in Equation 1 can be interpreted as the contribution of each contributing factor (z) to the concentration index (CI_H_), which consists of two parts. It is a product of the concentration index of each contributing factor (*Cj*) and the elasticity of *h*_*i*_ with respect to *z*_*j*_(i.e. $NGj=\;\beta_{j}{\bar{Z}}_{j}/\mu_{H}$). The unexplained portion is computed as the difference between CI_H_ and the deterministic portion. The residual cannot be systematically explained by variations in the contributing factors across socioeconomic groups (46). The generalised linear model (with binomial family and identity link) was applied in the decomposition analysis (47). Guided by variable availability in the dataset and well-established literature in the field (15, 24–26, 48, 49), determinants of care utilisation were separated into “need” (i.e. body mass index (BMI), age and sex for screening; age and sex only for treatment) and “non-need” (i.e. education level, exposure to media, employment status, rural or urban residence, and quintiles of SES) factors for both screening and treatment. A negative (positive) contribution suggests a given determinant contributes to inequality in the pro-poor (pro-rich) direction.

Given the challenge in computing analytical standard errors (SEs) for the components in the decomposition (i.e. elasticities and each contributing factor's contribution to the concentration index) in Equation 1, the bootstrap method (50, 51) was used to obtain such SEs in the analysis. The sampling structure of the data was taken into account as applied by Ataguba et al. (52) to avoid inconsistent estimates of bootstrap SEs. A total of 1,000 replications were used to estimate the SEs for each estimate.

### Measuring Horizontal Inequity in Care Utilisation

Horizontal equity analysis assesses inequity in care utilisation by standardising health service utilisation based on need (40). Inequity in care use estimated through the horizontal inequity (HI) index embodies the principle that healthcare should be utilised according to need (i.e. “equal treatment for equal need”). The HI was computed as the difference between the concentration index for actual (observed) care utilisation and need-expected utilisation. An indirect standardisation approach was used to predict the need-expected utilisation of screening and treatment (40, 53). HI lies within the range of −1 to +1, with a negative (positive) value indicating a pro-poor (pro-rich) inequity. Theoretically, a zero value for HI means there is no inequity. To estimate how much care each individual would receive if they were treated equally to everyone in the sample with equal needs, we fitted a regression model (40). All statistical analyses were conducted in Stata (version 15.1).

## Results

### Descriptive Analysis

Most respondents were female (60%). About 30% aged 20–39 years and 47% had attained primary education (Table 2). Only 19% of respondents were not employed, and more than half (54%) resided in a rural area. Hypertension prevalence was 30%, with a higher prevalence among wealthier individuals (Table 2). In addition, the prevalence of hypertension was higher among obese individuals (52%) compared to other BMI categories (underweight 19%, normal 26%, and overweight 39%) (data not shown). Although there was no significant difference, the prevalence of hypertension was slightly higher among non-smokers (30%) compared to non-smokers (29%) (data not shown).

**Table 2:** Descriptive statistics grouped by wealth quintile.

	**All**	**Household socioeconomic groups**					***p-*** value^*^
	**Col %**	**Poorest quintile**	**2nd quintile**	**3rd quintile**	**4th quintile**	**Richest quintile**	
**N**	4,500	918	891	899	909	883	
**Sex**							
Female	60.0	65.2	60.4	58.4	61.1	54.8	<0.01
Male	40.0	34.8	39.6	41.6	38.9	45.2	
**Age (years)**							
<19	4.5	5.0	5.5	4.4	4.2	3.5	<0.01
20–29	28.6	24.3	25.7	26.2	30.1	36.8	
30–39	28.0	28.7	29.4	27.9	24.6	29.2	
40–49	17.7	16.8	15.9	20.0	19.0	16.6	
50–59	12.1	13.5	13.1	12.0	12.8	8.9	
60+	9.2	11.6	10.5	9.5	9.3	5.0	
**Education level**							
No formal schooling	16.8	50.9	11.0	9.1	8.5	3.4	<0.01
Primary	46.5	40.0	63.3	59.1	45.3	24.9	
Secondary	25.5	8.2	22.0	25.5	33.0	39.1	
Tertiary	11.2	0.9	3.7	6.3	13.2	32.6	
**Marital status**							
Married/Cohabiting	67.9	72.6	65.2	68.2	70.0	63.1	<0.01
Not married	32.1	27.4	34.8	31.8	30.0	36.9	
**Exposure to media**							
Has TV/Radio	70.3	30.7	65.6	78.5	70.3	95.4	
No TV/Radio	29.7	69.3	34.4	21.5	29.7	4.6	<0.01
**Employment status**							
Unemployed	18.5	3.6	9.2	15.5	24.8	40.1	<0.01
Informal employment	39.7	33.9	43.0	43.9	39.4	38.4	
Formal employment	41.8	62.5	47.8	40.6	35.8	21.5	
**Residence**							
Rural	53.7	77.9	76.5	56.3	42.5	14.3	<0.01
Urban	46.3	22.1	23.5	43.7	57.5	85.7	
**BMI (Kg/m** ^**2**^ **)**							
<18.5 (underweight)	11.7	27.3	10.4	9.0	6.8	4.8	<0.01
18.5–24.9 (normal)	56.8	60.8	66.0	59.4	54.8	42.7	
25.0–29.9 (overweight)	21.0	9.5	17.6	22.6	22.8	32.6	
≥30.0 (obese)	10.5	2.4	6.0	9.0	15.6	19.9	
**Presence of NCD**							
Has hypertension	30.2	24.7	28.4	30.9	32.7	34.1	

* *p-value from Chi-squared test*.

### Inequality in Need and Use of Hypertension Screening and Treatment Services

**Figure 1:**
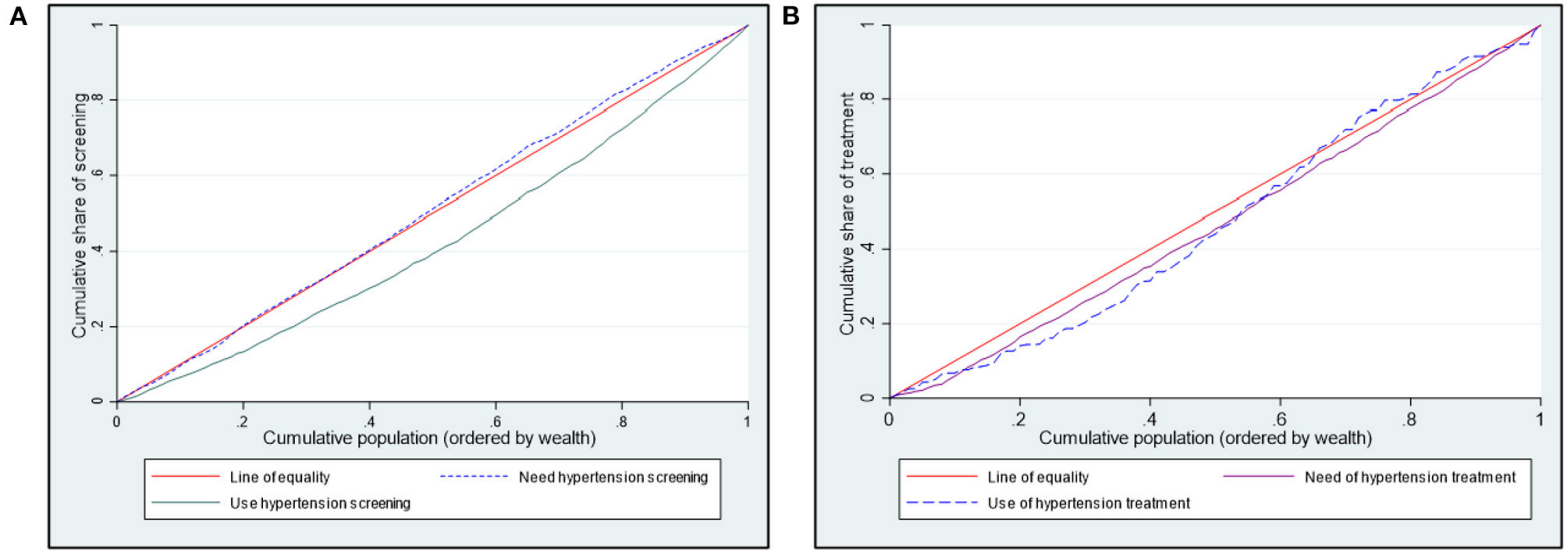
Concentration curves showing need and use of hypertension **(A)** screening and **(B)** treatment in Kenya.

Poorer individuals had a higher need for hypertension screening (concentration curves lie above the equality line), while hypertension screening favoured the rich (concentration curves lie below the equality line) (Figure 1). Although not significant, the need for hypertension screening was pro-poor (*CI* = −0.036; *p* > 0.05). On the other hand, hypertension screening (*CI* = 0.293; *p* < 0.01) was significantly pro-rich (Table 3).

**Table 3:** Concentration indices for need and use of screening services for hypertension in Kenya (STEPs 2015).

**Intervention**	**Concentration index**	**Std. Error**	***p-value***
Need hypertension screening	−0.036	0.027	0.186
Screening for hypertension	0.293	0.041	<0.001^**^
Need hypertension treatment	0.026	0.044	0.552
Use hypertension treatment	0.030	0.088	0.738

** *p < 0.001*.

**Figure 2:**
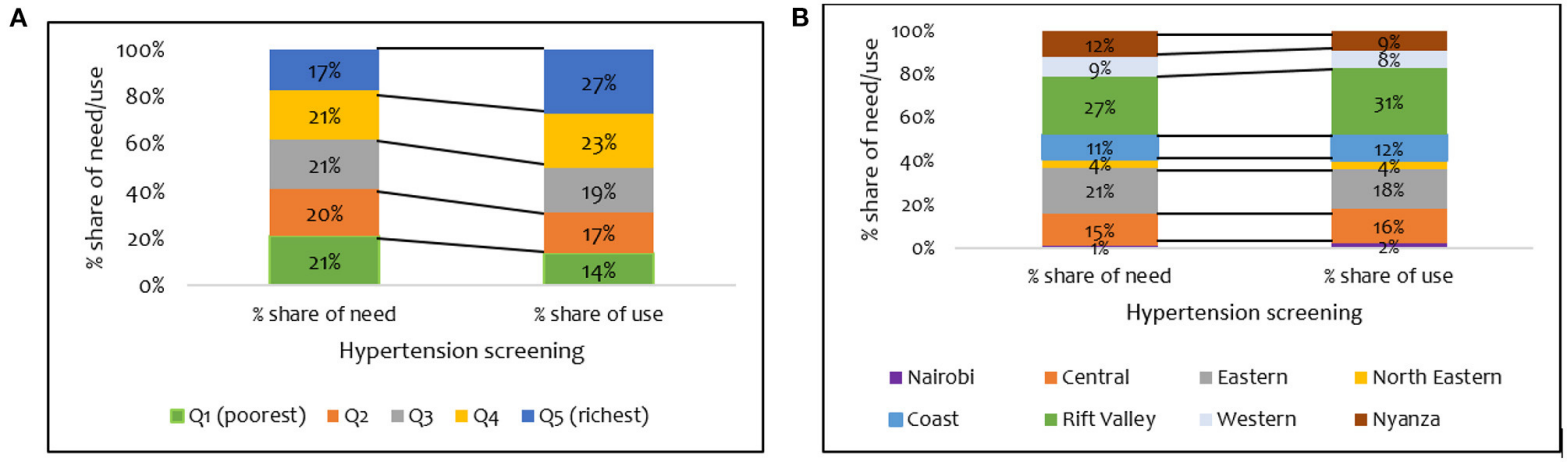
Distribution of share of need and use of hypertension screening services by **(A)** wealth quintile and **(B)** regions in Kenya (STEPs 2015).

A further breakdown of who benefits from hypertension screening revealed that the wealthier quintiles benefited disproportionately more than they should given their share of need (Figure 2A). For example, while only 17% of those needing hypertension screening are in the wealthiest quintile, 27% of individuals using screening interventions are in the wealthiest quintile (Figure 2A). There were disparities in the need and use of hypertension screening in all the regions (Figure 2B). For instance, the disparity between the share of need and use for hypertension screening was highest in the Rift Valley region (27% vs. 31%) (Figure 2B).

**Figure 3:**
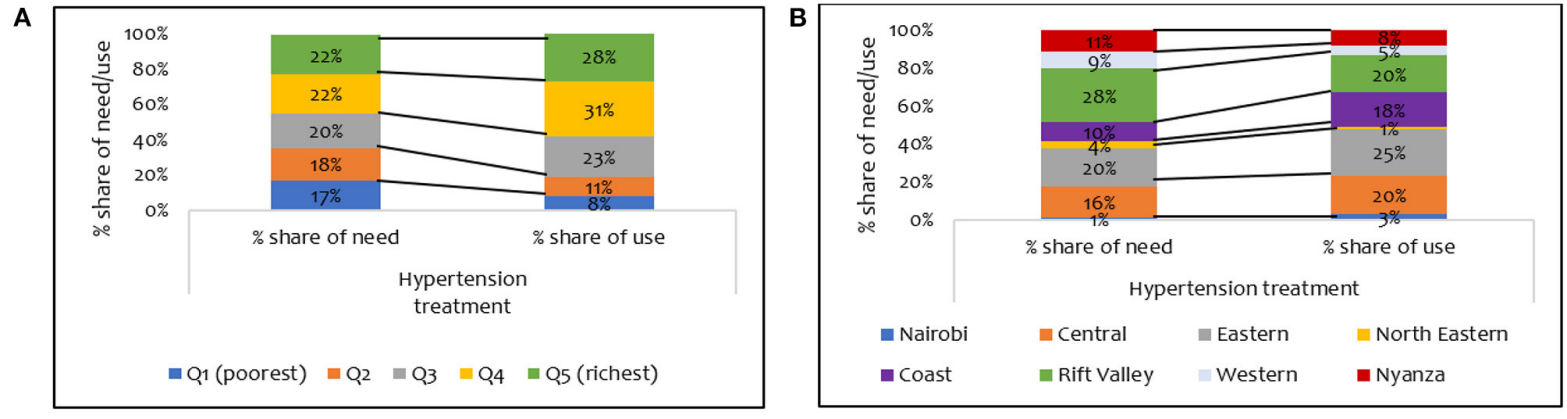
Distribution of share of need and use of hypertension treatment services by **(A)** wealth quintile and **(B)** regions in Kenya (STEPs 2015).

Figure 1B shows a pro-rich distribution of the need for hypertension treatment, a finding confirmed by the concentration indices in Table 3. The use of hypertension treatment was pro-rich (*CI* = 0.030; *p* > 0.05) (Table 3). However, none of the pro-rich or the pro-poor inequality findings for hypertension treatment was statistically significant at conventional levels. Individuals in the poorest quintile were disadvantaged in using hypertension treatment compared to their population share of need. For instance, while 17% of respondents needing hypertension treatment are in the poorest quintile, only 8% of those using hypertension treatment are in the poorest quintile (Figure 3A). Overall, a disproportionate share exists in using hypertension treatment in the Kenyan regions, given the population share of need. Notably, the disparity in the need and use of hypertension (28% vs. 20%) treatment was highest in the Rift Valley region (Figure 3B).

### Inequity in Using Screening and Treatment Services for Hypertension in Kenya

The use of hypertension screening was significantly pro-rich after controlling for need (*HI* = 0.185; *p*<*0.001*). Also, the use of hypertension treatment services was significantly pro-rich at conventional levels (Table 4).

**Table 4:** Inequity in the utilisation of screening and treatment services for hypertension in Kenya (STEPS 2015).

**Intervention**	**Horizontal equity index**
Hypertension screening	0.185^***^ (0.024)
Hypertension treatment	0.095^***^ (0.074)

*** *Standard errors reported in parentheses. p < 0.001*.

### Decomposition of Inequality in Care Use

**Figure 4:**
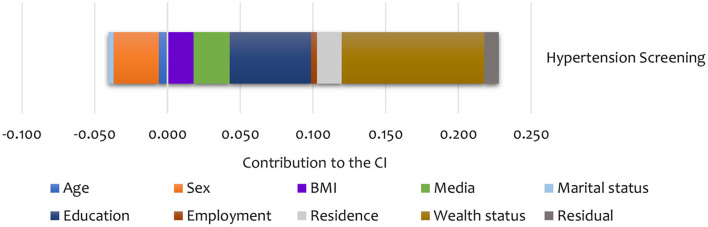
Summary contributions to SES inequality in the utilisation of screening services for hypertension in Kenya (STEPs 2015).

**Figure 5:**
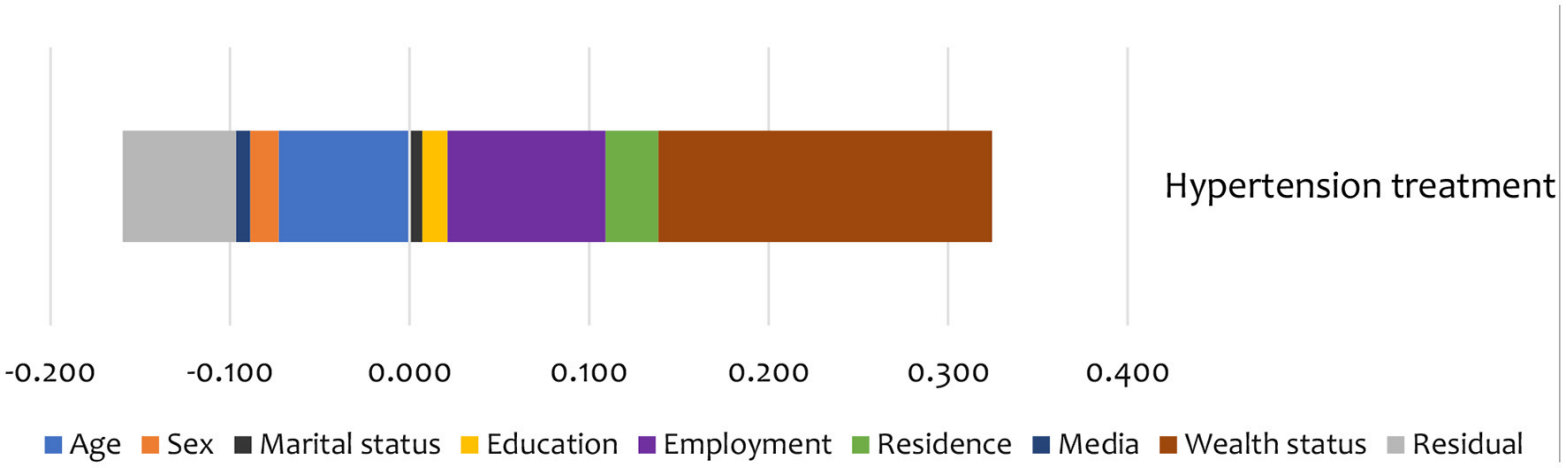
Summary contributions to SES inequality in the utilisation of treatment services for hypertension in Kenya (STEPs 2015).

Summary results of the decomposition analysis of inequality in screening and treatment are presented in Figures 4, 5, showing an aggregate contribution of need and non-need factors. In general, non-need factors contributed most to the pro-rich inequality in screening and treatment. Specifically, wealth status, exposure to media, education, and area of residence contributed most to inequality in screening among the non-need factors for hypertension. For the need factors, BMI explained inequality in the pro-rich direction for hypertension screening. However, sex explained inequality in the pro-poor direction for hypertension screening (Figure 4).

Non-need factors like wealth and employment status were the largest contributors (in the pro-rich direction) to the inequality in hypertension treatment (Figure 5). Additionally, age and education status also contributed to inequality in hypertension treatment in the pro-poor direction (Figure 5).

As shown in Table 5, sex was the main statistically significant contributor to inequality in hypertension screening among the need factors. Only a few categories are significant for some need factors like age and BMI. Among non-need factors, exposure to media was a statistically significant contributor (in the pro-rich direction) to inequality in hypertension screening. For hypertension treatment, age explained the inequality in the pro-poor direction. Among the non-need factors, wealth status explained, to a greater extent, inequality in hypertension (in the pro-rich direction) treatment (Table 5). However, none of the need and non-need contributors to inequality hypertension treatment was statistically significant at conventional levels.

**Table 5:** Decomposition analysis of inequality in the utilisation of screening and treatment services for hypertension in Kenya (STEPs 2015).

	**Hypertension screening**					**Hypertension treatment**				
**Determinants**	**Elasticity**	**CI**	**Contribution**	**Total contribution**	**% contribution**	**Elasticity**	**CI**	**Contribution**	**Total contribution**	**% Contribution**
**BMI**										
Underweight (Ref)										
Normal	−0.004	−0.155^***^ (0.014)	0.001 (7.976)	0.018	6.068					
Overweight	0.026	0.255^***^ (0.032)	0.007 (0.004)							
Obese	0.025	0.398^***^ (0.036)	0.010^**^ (0.002)							
**Sex**										
Male (Ref)										
Female	0.400	−0.078^**^ (0.017)	−0.031^**^ (0.006)	−0.031	−11.023	0.203	−0.078^**^ (0.044)	−0.016 (1.110)	−0.016	−19.231
**Age**										
<19 (Ref)										
20–29	0.073	0.115^***^ (0.025)	0.008 (4.332)	−0.006	−1.666	−0.854	0.115(0.109)	−0.098(2.944)	−0.073	−89.802
30–39	0.095	−0.019^**^ (0.026)	−0.002 (0.267)			−0.511	−0.019(0.084)	0.001(1.264)		
40–49	0.085	0.024 (0.028)	0.002 (0.003)			−0.220	0.024(0.062)	−0.005(0.016)		
50–59	0.058	−0.083 (0.044)	−0.005 (0.010)			−0.148	−0.083(0.062)	0.012(1.000)		
60+	0.063	−0.135^**^ (0.058)	−0.009^**^ (0.004)			−0.060	−0.135(0.088)	0.008(0.013)		
**Exposure to media**										
No TV/Radio (Ref)										
TV/Radio	0.050	0.531^***^ (0.023)	0.027^**^ (0.004)	0.027	10.891	−0.021	0.384^***^ (0.000)	−0.008 (0.997)	−0.008	−26.667
**Marital status**										
Married (Ref)										
Not married	−0.060	0.058^**^ (0.026)	−0.004 (37.064)	−0.004	−1.226	0.124	0.058 (0.079)	0.007 (0.151)	0.007	18.342
**Residence**										
Urban (Ref)										
Rural	−0.032	−0.539^***^ (0.043)	0.017 (3.243)	0.017	6.014	−0.055	0.539^***^ (0.064)	0.029 (3.851)	0.029	35.877
**Employment**										
Not employed. (Ref)										
Informal employment	−0.011	0.021 (0.027)	0.000 (0.476)	0.011	3.548	−0.173	−0.081 (0.0978)	−0.014 (0.455)	0.021	25.520
Formal emp.	0.029	0.392^***^ (0.035)	0.011 (0.017)			−0.082	0.434^***^ (0.044)	0.035 (8.105)		
**Education**										
No school (Ref)										
Primary school	0.074	−0.149^***^ (0.033)	−0.011 (7.858)	0.056	19.905	−0.251	−0.149^***^ (0.061)	0.019 (0.425)	0.014	46.667
Secondary school	0.079	0.311^***^ (0.029)	0.025 (298.5)			−0.221	0.320 (0.084)	−0.019 (0.587)		
Tertiary	0.067	0.621^***^ (0.042)	0.042^***^ (0.007)			−0.069	0.621^***^ (0.050)	0.014 (0.896)		
**Wealth quintile**										
Quintile 1 (Ref)										
Quintile 2	0.026	−0.491^***^ (0.055)	−0.013 (153.8)	0.098	34.731	0.007	0.491^***^ (0.059)	−0.004 (0.305)	0.186	227.125
Quintile 3	0.037	0.005 (0.069)	0.000 (0.096)			0.118	0.005^**^ (0.083)	0.001 (0.148)		
Quintile 4	0.052	0.508^***^ (0.073)	0.026 (0.247)			0.196	0.508 (0.098)	0.010 (0.055)		
Quintile 5	0.084	1.000^***^ (0.038)	0.084^***^ (0.017)			0.090	1.000^***^ (0.053)	0.090 (11.462)		
Residual			0.057	0.057	35.758				−0.296	−117.831

Bootstrapped standard errors in parentheses using 1,000 replications; CI, concentration index; *p < 0.1;

** *p < 0.05;*

*** *p < 0.01*.

## Discussion

This study demonstrated the existence of socioeconomic inequality and horizontal inequity in the use of screening and treatment interventions for hypertension in Kenya. These findings can serve as a baseline for future progress assessments towards attaining SDG 3.4 targeted at NCDs and UHC goals in Kenya. In general, the results confirm that need does not match the use of screening and treatment services for hypertension across the SES groups and the Kenyan regions.

This paper's findings add to the evidence that the Kenyan health system is unequal and inequitable (54–56). It suggests that policy interventions geared towards attaining equity in the Kenyan health system should pay special attention to NCDs like hypertension. Among other policy options, it has been established that timely screening among those at risk and treatment among those diagnosed are cost-effective strategies for combating the burden of hypertension (10, 11). However, our findings reveal considerable gaps in meeting the population need for both interventions in Kenya.

Poorer socioeconomic groups need more hypertension screening than their wealthier counterparts, but wealthier socioeconomic groups benefit more from screening services than their share of need. This finding could, in part, be explained by broader access barriers such as availability (i.e. biased availability of health facilities in urban locations), acceptability (i.e. providers and patients attitudes and expectations of each other) and affordability of screening services (57). For instance, transport costs have been shown to not only lead to catastrophic expenditures for hypertension treatment in public health facilities in Kenya but also contribute to about 40% of total out-of-pocket costs (58). Furthermore, the unaffordability of NCDs screening services in Kenya has been documented, with healthcare costs being disproportionately higher in the private relative to the public sector (59). Sex was the primary “need” factor contributing to socioeconomic inequality in hypertension screening, suggesting that females are more likely to seek screening services for hypertension. A similar finding has been reported in a study that assessed socioeconomic inequality for diabetes and hypertension screening in South Africa (25). Of interest, being obese was the other significant contributor (in the pro-rich direction) to socioeconomic inequality in hypertension screening (Table 5). Given that being obese is a risk factor for cardiovascular diseases (27), this finding suggests that those who are wealthy and obese are more likely to utilise hypertension screening services compared to other BMI categories. Also, given that “non-need” determinants such as exposure to media and education contributed to inequality in the screening for hypertension in the pro-rich direction, unawareness of the importance of timely or early screening may provide some insights into the possible reasons for the underutilisation of screening services among poorer socioeconomic groups.

Whereas the geographic spread of health facilities in Kenya has increased over time (60), other supply-side factors such as the physical inaccessibility of health facilities remain a barrier for using screening services as the area of residence contributed to pro-rich inequality. Also, the weak health system capacity to offer care for NCDs, particularly at the primary care level, could explain the inequality in hypertension screening (61, 62). A fragmented health service delivery structure biased towards offering curative rather than preventive healthcare services is among examples of health system weaknesses (63–65). The inequality and inequity in the screening for hypertension compare well with the findings of a South African study that has shown marked pro-rich inequality and inequity in the screening for hypertension, diabetes and cholesterol, with non-need factors (i.e. wealth status, health insurance, and education) mainly contributing to the inequality (25).

This study also found that hypertension treatment needs do not match how different SES groups use the service. The wealthier quintiles relative to the poorer ones benefited more than their treatment needs, and this disparity existed in the Kenyan regions. Among other things, this finding can be explained by the overall unaffordability of NCDs services in Kenya (59) and the low levels of health insurance coverage (66), with lower socioeconomic groups being disproportionately affected. Similar patterns have been reported in previous studies in Kenya that have assessed inequality in health and healthcare use or access at the sub-national level (24, 54–56). While inequality in the use of hypertension treatment was not statistically significant, the horizontal inequity finding suggests that hypertension treatment is not distributed according to need. Besides, compared to the inequality observed in hypertension screening, the “relative equality” in hypertension treatment could be partly explained by the difference in distribution of the burden of hypertension in the Kenyan population (i.e. 42% of rural dwellers have hypertension while 30% of urban dwellers have hypertension). Therefore, it can be argued that the pro-rich distribution in the use of hypertension treatment services could be as a result of the pro-urban bias in the distribution of healthcare facilities in Kenya and thus the rural dwellers are disadvantaged at utilising hypertension treatment services while they are most in need (60). Of note, the findings compare well with previous multi-country studies that found significant pro-rich inequality in the use of secondary cardiovascular medicines in LMICs (17) and hypertension treatment (32).

In the decomposition analysis of inequality in hypertension treatment, non-need factors primarily contributed to the observed inequality. For instance, area of residence, wealth, employment and education status contributed to inequality in hypertension treatment. Similar findings have been reported in China, where non-need factors such as income, area of residence, longest-held occupation, and level of education were significant contributors to the socioeconomic inequality in the utilisation of hypertension, hyperglycaemia and dyslipidaemia treatment (15). Unlike findings from other settings (18, 26), in this study, hypertension prevalence was higher among wealthier socioeconomic groups than their poorer counterparts. This disproportionate hypertension burden may lead to differences in healthcare demand between the rich and the poor in Kenya.

Several policy recommendations are imperative from the findings of this study. First, since service delivery falls within the docket of county governments in Kenya, there is an urgent need to enhance the capacity of primary care facilities to implement cost-effective strategies such as timely screening so that need can match service use for this critical intervention. Second, for demand in the utilisation of screening services to be increased, national and county governments, including other relevant actors, should implement strategic awareness-raising campaigns targeting at-risk populations as age and sex contributed to the SES inequality in the screening of hypertension. This can be through targeted health education messages in the mass media and other appropriate channels. Third, while recent efforts by the government of Kenya to attain UHC by 2022 are timely and commendable, more needs to be done to ensure the realisation of equity in the use of NCD services. Given the interplay of factors beyond the health sector that affect health, as was seen in the role of non-need factors in contributing to inequality in screening and treatment, there is a need for multi-sectoral approaches at various levels (i.e. local, national and regional) to address drivers of poverty and social inequity with a critical focus in marginalised areas. Some of the sectors that could collaborate with the health sector in addressing inequities/inequalities in NCDs include education and media. For instance, for increased health education on NCDs, the education sector can include NCDs in the curriculum. Also, various media channels can be used to raise awareness on the benefits of early NCDs screening as exposure to media was shown to contribute to SES inequality in hypertension screening.

## Study Strengths and Limitations

This study had strengths and limitations. One key strength was the national representativeness of the data set used, which gave the national picture of socioeconomic inequality and inequity in the screening and treatment for hypertension in Kenya. Also, while previous studies have mainly assessed inequalities in the prevalence of NCDs (and in most cases using self-reported data), this study examines inequity and socioeconomic inequality in key interventions using objective measures of need for screening and treatment. This study also used a novel methodological approach: the decomposition analysis, to uncover factors contributing to socioeconomic inequality in screening and treatment for hypertension in Kenya.

This study also had limitations. The first limitation was data-driven. As is common in studies on care utilisation, we relied on self-reported data in defining the use of both screening and treatment. This could potentially bias our inequality findings, especially if there were cases of misreporting. Likewise, although previous studies (67, 68) have reported no association between under-reporting of care utilisation and demographic characteristics, except for age, we cannot rule out under-reporting of care utilisation in the low SES groups. Second, the STEPs data set was cross-sectional in design and thus limiting the establishment of temporal trends in inequality and inequity in the use of screening and treatment. Furthermore, it is essential to note that causality is not implied for the factors explaining observed inequality in screening and treatment.

## Conclusion

Kenya faces a rising disease burden from non-communicable diseases, as expected in many low-and middle-income countries. This paper provides the first empirical evidence on socioeconomic inequality and inequity in screening and treatment interventions for NCDs based on need in Kenya. These findings provide a benchmark for future equity and equality assessments for NCDs in Kenya. In keeping with the global UHC agenda and other key NCDs targets, there is an urgent need for concerted efforts to ensure equity in providing NCDs healthcare services in Kenya. Indeed, given the ongoing policy reforms to attain UHC in Kenya, a window of opportunity exists to avert inequity in NCDs, with this paper highlighting some of the critical issues for consideration.

## Data Availability Statement

The raw data supporting the conclusions of this article will be made available by the authors, without undue reservation.

## Ethics Statement

The studies involving human participants were reviewed and approved by Human Research Ethics Committee of the University of Cape Town (Ref: 186/2020). The patients/participants provided their written informed consent to participate in this study.

## Author Contributions

RO, JA, and EB conceived the study. RO conducted the data analysis and wrote the first draft of the manuscript. All authors have read and agreed to the published version of the manuscript.

## Funding

The authors acknowledge funding support from the Wellcome Trust International Masters Fellowship (Grant Award Number: 214622) awarded to RO. EB is funded by a Wellcome Trust core grant (#092654).

## Conflict of Interest

The authors declare that the research was conducted in the absence of any commercial or financial relationships that could be construed as a potential conflict of interest.

## Publisher's Note

All claims expressed in this article are solely those of the authors and do not necessarily represent those of their affiliated organizations, or those of the publisher, the editors and the reviewers. Any product that may be evaluated in this article, or claim that may be made by its manufacturer, is not guaranteed or endorsed by the publisher.

## Footnotes

^1^UHC aims to ensure utilisation of quality healthcare services by everyone based on need and without suffering financial hardship.
